# Post-cardiac arrest mortality is declining in the UK

**DOI:** 10.1186/s13054-016-1481-4

**Published:** 2016-09-29

**Authors:** Saket Girotra

**Affiliations:** Division of Cardiovascular Diseases, Department of Internal Medicine, University of Iowa Carver College of Medicine, 200 Hawkins Drive, Suite 4427 RCP, Iowa City, IA 52242 USA

Cardiac arrest is a leading cause of death in the developed world. In the US, more than 500,000 patients die from a cardiac arrest every year [1]. The public health impact of cardiac arrest in the UK is also quite substantial [2]. While mortality is high, even those who survive a cardiac arrest are at a significant risk of permanent neurological damage. Thus, improving survival and reducing neurological disability is a fundamental goal of patient care and a priority for public health.

Conceptually, mortality in cardiac arrest occurs in two distinct phases. Patients either die acutely due to a failure to attain return of spontaneous circulation (ROSC), which depends on timely, high-quality resuscitation, or, alternatively, they die following ROSC due to a cascade of clinical events, which may depend on invasive treatments, quality of nursing, and critical care (post-resuscitation phase). In recent years, the post-resuscitation phase has been recognized as an important phase of resuscitation. The European Resuscitation Council and the American Heart Association have published guidelines emphasizing treatment strategies for optimizing post-resuscitation care, which includes early coronary angiography, targeted temperature management (TTM), close monitoring and treatment in intensive care unit (ICU), delaying neurological prognostication, and regionalizing care [3, 4]. In its recent report *Strategies to Improve Cardiac Arrest Survival* the Institute of Medicine included improving systems of resuscitation care within hospitals as a key recommendation to improve outcomes [5].

In the current issue of *Critical Care*, Professor Nolan and colleagues provide interesting new data regarding temporal patterns of post-resuscitation management and outcomes in the UK. Using data from a national ICU audit, 63,417 patients across 286 ICUs who had received cardiopulmonary resuscitation and mechanical ventilation within 24 h were included. A substantial reduction in in-hospital mortality over an 11-year period was noted—both in patients with out-of-hospital cardiac arrest (OHCA: 70.1 % in 2004 to 66.4 % in 2014; risk-adjusted odds ratio [OR] per year 0.96; 95 % confidence interval [95 % CI] 0.95–0.97, *P* value for trend <0.001) and in-hospital cardiac arrest (IHCA: 70.4 % in 2004 to 60.3 % in 2014; risk-adjusted OR per year 0.96, 95 % CI 0.95–0.97, *P* value for trend <0.001). Risk adjustment in the study was performed using a validated hierarchical model that accounted for case mix and within-ICU clustering. A decline in post-cardiac arrest mortality was accompanied by an increase in use of therapeutic hypothermia, indirectly measured as the lowest temperature of <34 °C and avoidance of early treatment withdrawal. The rate of organ donation among non-survivors also increased threefold (3.1 % in 2004 to 10.1 % in 2014), providing a needed impetus to the low rates of organ donation in the UK [6].

The study has a few limitations, many of which were acknowledged by the authors. First, although large and comprehensive, data collection was not based on the Utstein template and important variables, such as initial rhythm, were not available. Given that cardiac arrests due to ventricular rhythms, which are associated with significantly better survival, have declined in recent years [7, 8], it is possible that the survival trends noted in the current study are even more pronounced. Second, data on neurological function in survivors were also not available, which makes it difficult to assess whether the substantial improvement in survival was clinically meaningful. Third, patients who experienced IHCA while already admitted and receiving care in an ICU were not included, a group that comprises over 50 % of IHCA cases in the US [8].

Despite the above limitations, Nolan and colleagues need to be congratulated for providing detailed information regarding post-cardiac arrest outcomes in a large cohort of patients in the UK. Their findings add to a growing body of literature that has shown an unmistakable improvement in survival and neurological outcomes for both OHCA and IHCA [8, 9]. Such data challenge the existing nihilism associated with resuscitation and can potentially invigorate research and quality improvement efforts to ensure that improved outcomes are sustained over time.

So, what is the mechanism of mortality reduction noted in this study? The authors attributed it to increasing use of hypothermia and delaying treatment withdrawal, strategies emphasized in the guidelines [3, 4]. However, causal interpretation is difficult due to the design of this study. Although recommended in all comatose survivors of cardiac arrest, the benefit of hypothermia has only been demonstrated in patients with OHCA due to ventricular rhythms [10, 11]. Among patients with asystole or pulseless electrical activity, or patients with IHCA, the benefit of hypothermia is not proven [12]. Moreover, even among patients with OHCA, recent data from the TTM trial have shown that cooling to 36 °C provided the same benefit as more intensive cooling to 33 °C [13].

Accurate timing of neurological prognostication also remains a challenge in clinical practice. Current guidelines recommend delaying neurological prognostication for 72 h or longer after achieving normothermia to avoid premature treatment withdrawal. Over the study period, there was a notable increase in the median time between cardiac arrest and time of treatment withdrawal from 2.5 to 3.3 days. While data were not stratified by hypothermia treatment, it is conceivable that early withdrawal of care before the recommended time period does occur in some patients. However, this reflects the reality of clinical practice where physicians must balance the risk of providing care that is perceived as unnecessary or futile with the risk of premature withdrawal while managing expectations of family members and respecting patients’ stated wishes.

Although the above trends in hypothermia and timing of care withdrawal are noteworthy, it isn’t clear whether they are the sole drivers of survival improvement. Post-resuscitation survival depends on a multitude of factors, such as timely provision of life-saving treatments, multi-disciplinary teams, comprehensive critical/nursing care, and continuous quality improvement, all of which are enabled by institutional expertise, resources, and leadership. An important next step may be to examine site-level variation in survival to identify sites that consistently achieve exceptional post-cardiac arrest outcomes [14]. Identifying and evaluating processes employed by such outlier sites, using a combination of quantitative and qualitative methods could help identify best practices for post-resuscitation care [15]. Implementation of best practices across sites could ensure that the survival trends noted in this study are sustained over time.

## Abbreviations

CI: Confidence interval
ICU: Intensive care unit
IHCA: In-hospital cardiac arrest
OHCA: Out-of-hospital cardiac arrest
OR: Odds ratio
TTM: Targeted temperature monitoring
